# Optimal immediate sagittal alignment for kyphosis in ankylosing spondylitis following corrective osteotomy

**DOI:** 10.3389/fsurg.2022.975026

**Published:** 2022-09-05

**Authors:** Jianzhou Luo, Kai Yang, Zili Yang, Chaoshuai Feng, Xian Li, Zhenjuan Luo, Huiren Tao, Chunguang Duan, Tailin Wu

**Affiliations:** ^1^ Department of Orthopaedics, Shenzhen University General Hospital, Shenzhen, China; ^2^ Department of Orthopaedics, Xi’an Red Cross Hospital, Xi’an, China; ^3^ The Key Laboratory of Biomedical Information Engineering of Ministry of Education, School of Life Science and Technology, Xi'an Jiaotong University, Xi'an, China

**Keywords:** ankylosing spondylitis, kyphosis, optimal sagittal alignment, osteotomy, clinical outcome

## Abstract

**Purpose:**

To investigate the optimal immediate sagittal alignment of kyphosis in ankylosing spondylitis (AS) following corrective osteotomy.

**Methods:**

Seventy-seven AS patients who underwent osteotomy were enrolled. Radiographic parameters, including global kyphosis (GK), lumbar lordosis (LL), T1 spinopelvic inclination (T1SPI), sagittal vertical axis (SVA), T1 pelvic angle (TPA), pelvic incidence (PI), pelvic tilt (PT), sacral slope (SS), and PI and LL mismatch (PI–LL), were collected. The clinical outcome was evaluated using the Scoliosis Research Society-22 (SRS-22) questionnaire and Oswestry Disability Index (ODI). At the final follow-up, SVA > 5 cm was regarded as sagittal imbalance, and a total ODI ≤ 20 or total SRS-22 score ≥4 was considered to indicate a good clinical outcome.

**Results:**

Seventy-seven patients with an average age of 37.4 ± 8.6 years were followed up for 29.4 ± 4.2 months. At the final follow-up, GK, LL, PT, SS, TPA, and T1SPI showed some degree of correction loss (*P *< 0.05). The follow-up parameters could be predicted with the immediate postoperative parameters through their linear regression equation (*P *< 0.05). The postoperative immediate T1SPI, TPA, SVA, and PI were also highly correlated with the clinical outcome (ODI and/or SRS-22) at the final follow-up (*P *< 0.05). Based on the relationship, the optimal immediate sagittal alignment for obtaining good clinical outcome was determined: T1SPI* *≤* *0.9°, TPA* *≤* *31.5°, and SVA* *≤* *9.3cm. AS patients with PI* *≤* *49.2° were more likely to achieve the optimal alignment and obtained lower ODI and a lower incidence of sagittal imbalance than those with PI > 49.2° at the final follow-up (*P *< 0.05).

**Conclusion:**

Postoperative immediate parameters could be used to predict the final follow-up parameters and clinical outcome. The optimal postoperative immediate sagittal alignment of AS patients was T1SPI* *≤* *0.9°, TPA* *≤* *31.5°, and SVA* *≤* *9.3 cm, providing a reference for kyphosis correction and a means for clinical outcome evaluation. Patients with a lower PI (≤49.2°) were more likely to achieve optimal alignment and obtain satisfactory clinical outcomes.

## Introduction

Ankylosing spondylitis (AS) is a chronic inflammatory disease. In advanced stages, AS is usually complicated by total spinal stiffness, thoracolumbar kyphosis, and pelvic retroversion. Individuals with AS might have trouble looking horizontally, lying flat, and standing or walking upright, seriously impairing their daily activities and quality of life (1). Osteotomy is the only effective method for correcting these deformities and providing these individuals with the opportunity for a normal life. However, for AS patients with kyphosis, the correction standard is elusive and the optimal postoperative immediate sagittal alignment following osteotomy is still not well understood (2).

Schwab et al. (3) proposed a sagittal vertical axis (SVA) <5.0 cm, pelvic tilt (PT) <25°, and pelvic incidence and lumbar lordosis mismatch (PI–LL) = ±9° as realignment objectives for adult spinal deformity (ASD). However, these values are different in AS patients. Recently, Huang et al. (4) reported PT < 24°, spinosacral angle >108°, T1 pelvic angle (TPA) < 22°, and spinopelvic angle >152° as the optimal sagittal objectives for AS patients at the 2-year follow-up. Notably, Huang proposed these values as goals for the 2-year follow-up; however, the optimal values for immediate alignment after surgery are still unknown and could be more pragmatic and meaningful for guiding kyphosis correction.

Therefore, in this study, we (1) determined the relationship between the immediate postoperative parameters and final follow-up parameters and clinical outcome in AS patients, (2) investigated an optimal immediate sagittal alignment based on the relationship, and (3) clarified the influence of PI on sagittal alignment and clinical outcome at the final follow-up.

## Materials and methods

### Subjects

Consecutive AS patients who underwent modified three-column osteotomy from January 2010 to July 2019 were reviewed retrospectively. The inclusion criteria were as follows: (1) age between 18 and 65 years old, (2) global kyphosis (GK) over 50°, (3) completed radiographs and clinical outcome measurements, and (4) a minimum of 2 years of follow-up. Patients with a history of previous spinal surgery, ankylosed hip or knee joints, postoperative pseudarthrosis, or instrumentation failure during the follow-up were excluded. Finally, a total of 77 AS patients, including 57 who underwent one-level osteotomy and 20 who underwent two-level osteotomy, met the criteria and were enrolled in this study.

The study was performed in accordance with the Declaration of Helsinki and approved by the institutional review board of Shenzhen University General Hospital. Informed consent was obtained from all participants before surgery.

### Data collection

**Figure 1:**
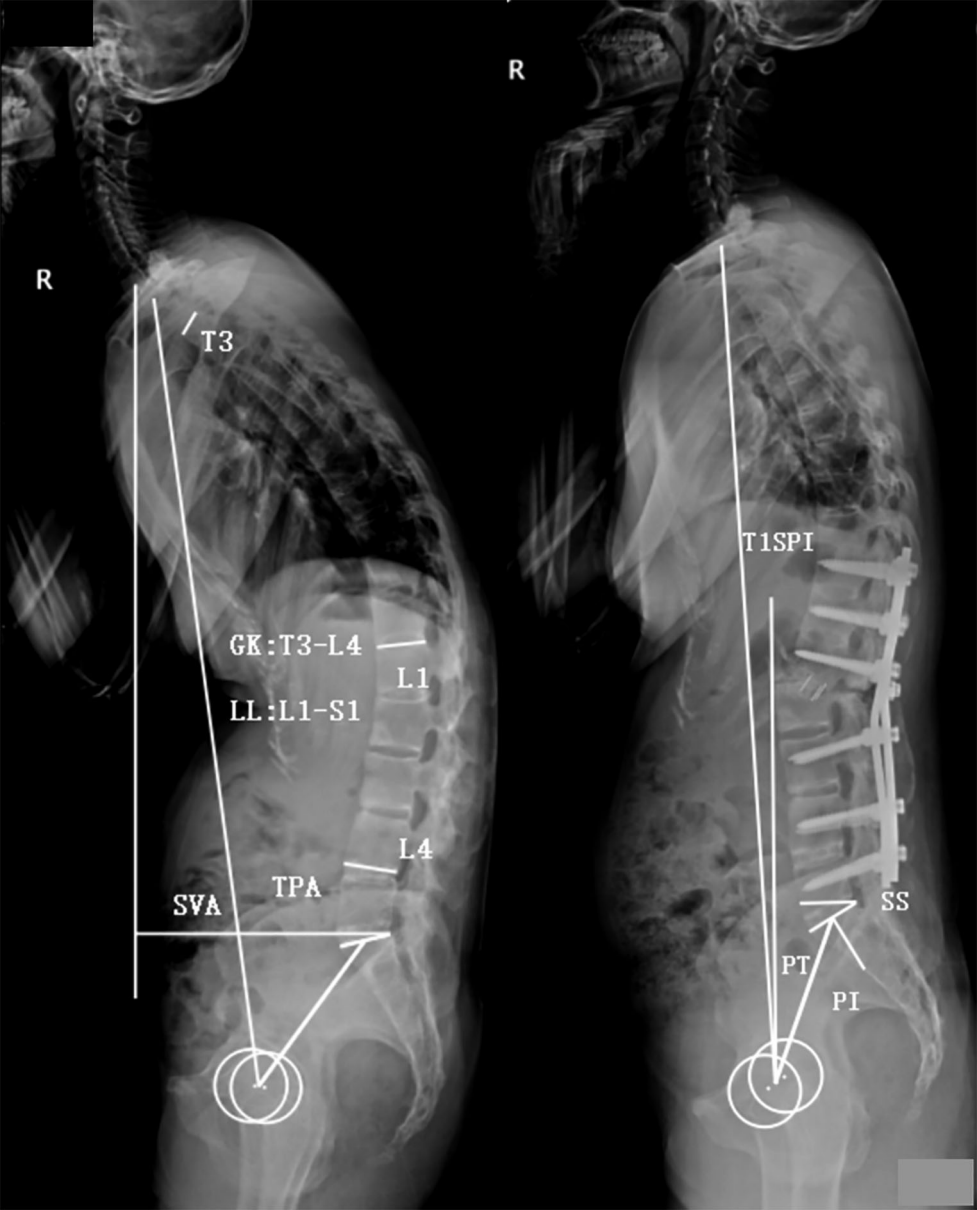
Illustration of radiographic parameters. Global kyphosis (GK): the angle between the superior endplate of the maximally tilted upper-end vertebra and the inferior endplate of the maximally tilted lower-end vertebra. Lumbar lordosis (LL): the Cobb angle from the L1 upper endplate to the S1 upper endplate (negative number represents lordosis, and positive number represents kyphosis). Sagittal vertical axis (SVA): the distance between the C7 plumb line and the posterior-superior corner of S1. T1 pelvic angle (TPA): the angle between a line joining the center of T1 and the femoral head axis and a line from the center of the femoral head axis to the midpoint of the S1 upper endplate. T1 spinopelvic inclination (T1SPI): the angle between the vertical line and a line from the center of the femoral head axis to the center of the T1 vertebral body (a negative number represents that T1 is posterior to the femoral head, and a positive number represents that T1 is anterior to the femoral head). Pelvic tilt (PT): the angle between the vertical line and the line from the center of the S1 upper endplate to the center of the femoral head axis. Pelvic incidence (PI): the angle between the perpendicular line to the S1 upper endplate and the line from the center of the S1 upper endplate to the center of the femoral head axis. Sacral slope (SS): the angle between the S1 upper endplate and the horizontal line. Pelvic incidence and lumbar lordosis mismatch (PI–LL): pelvic incidence value minus lumbar lordosis value.

Standing anteroposterior and lateral radiographs of the whole spine were obtained preoperatively, immediately postoperatively (3–4 weeks after surgery), and at the final follow-up (a minimum of 2 years after surgery). Several parameters were measured using lateral radiographs, including GK, LL, SVA, T1 spinopelvic inclination (T1SPI), TPA, pelvic incidence (PI), PT, sacral slope (SS), and PI–LL (Figure 1). The clinical outcome was evaluated using the Scoliosis Research Society-22 (SRS-22) questionnaire and Oswestry Disability Index (ODI). Data were collected before and after surgery as well as at the final follow-up. At the final follow-up, an SVA of >5 cm was regarded as sagittal imbalance (3), and a total ODI ≤ 20 or total SRS-22 score ≥4.0 was considered to indicate a good clinical outcome (4–6).

### Surgical technique

The modified three-column osteotomy was usually performed in the apical region of kyphosis so that substantial kyphosis correction could be achieved. The whole procedure was performed under somatosensory-evoked potential and motor-evoked potential monitoring. The resection area of the corrective osteotomy included the spinous process, the upper part of the lamina and superior articular processes of the osteotomized vertebra, as well as the lower part of the lamina and inferior articular processes of the cranially adjacent vertebra. The transverse process of the osteotomized vertebra was exposed and resected; then, subtotal resection was performed along the upper part of the pedicles to the front of the vertebral body, which usually involved resecting 1/3–1/2 of the upper part of the vertebral body together with the cranially adjacent intervertebral disc. The lower half of the vertebral pedicle, part of the lamina, and the intact inferior articular processes of the osteotomized vertebra were preserved.

Temporary rods were implanted after finishing the osteotomy. Before correction, the spinal cord was slightly shortened in advance by compressing the rods, with the aim of preserving the space for potential spinal cord lengthening during correction. Sequentially, kyphosis was corrected gradually by lifting up the patient's shoulders while simultaneously bending the rods. After achieving satisfying correction, the temporary rods were replaced with precontoured rods successively. Subsequently, a local bone graft and a cage filled with autogenetic bone were implanted sequentially in the osteotomy space, further compressing the rods. The bone autograft was spread on the surface of the lamina to facilitate spinal fusion. Postoperatively, the patients were allowed to ambulate with a customized thoracolumbosacral orthosis 3 days after surgery, which was typically maintained for 6 months.

### Statistical analysis

Measurement data are expressed as the mean ± standard deviation. Statistical analysis was performed using SPSS software (version 22.0, SPSS, Inc., Chicago, IL, USA). Paired *t*-tests were used to compare differences in the radiographic parameters and clinical outcome before and after surgery as well as at follow-up. The relationship between postoperative immediate parameters and final follow-up parameters was assessed with linear regression analysis. Correlations between postoperative immediate parameters and clinical outcome were evaluated using Pearson's correlation coefficient. Optimal thresholds of clinically relevant parameters were evaluated using receiver operating characteristic (ROC) curve analysis. Multiple stepwise linear regression analysis was performed to determine the key clinically relevant parameter and establish a predictive model for the total SRS-22 score. The difference between groups which was divided by the threshold of PI was using two independent *t*-tests and *χ*^2^ tests. A difference with a *P-*value <0.05 was considered statistically significant.

## Results

### General data

Seventy-seven AS patients (67 men and 10 women) with an average age of 37.4 ± 8.6 years (range, 20–64 years) and an average follow-up of 29.4 ± 4.2 months (range, 24–84 months) were included. The postoperative immediate GK, LL, PT, SS, PI–LL, TPA, T1SPI, and SVA were significantly improved (*P *< 0.01). At the final follow-up, GK, LL, PT, SS, TPA, and T1SPI showed some degree of correction loss (*P *< 0.05). Although the difference was not statistically significant, PI–LL (*P *= 0.078) and SVA (*P *= 0.115) also showed some loss of correction at the final follow-up. There was no significant difference in PI before and after surgery (*P *> 0.05) (Table 1).

**Table 1:** Differences of radiographic parameters in ankylosing spondylitis patients after surgery.

Parameters	Preoperative	Postoperative immediate	Final follow-up	Loss of correction
GK (°)	84.7 ± 24.8	32.9 ± 15.4**	35.1 ± 16.7	2.2 ± 6.8*
LL (°)	5.9 ± 21.9	−33.8 ± 17.2**	−31.7 ± 17.6	2.2 ± 7.6*
PT (°)	38.6 ± 11.5	28.5 ± 9.7**	31.0 ± 8.8	3.3 ± 5.2*
PI (°)	48.9 ± 13.5	48.5 ± 12.0	48.0 ± 11.2	0.5 ± 4.3
SS (°)	10.3 ± 12.8	20.2 ± 11.7**	15.8 ± 13.0	4.6 ± 8.5*
PI–LL (°)	53.9 ± 21.8	15.0 ± 15.4**	16.7 ± 16.4	1.7 ± 7.0
TPA (°)	57.9 ± 19.4	30.1 ± 11.6**	32.5 ± 10.9	1.7 ± 4.6*
T1SPI (°)	18.5 ± 16.8	1.8 ± 5.8**	0.3 ± 5.8	1.5 ± 4.8*
SVA (cm)	23.0 ± 9.0	9.5 ± 5.5**	8.6 ± 5.8	0.9 ± 4.1

GK, global kyphosis; LL, lumbar lordosis; PT, pelvic tilt; PI, pelvic incidence; SS, sacral slope; PI–LL, pelvic incidence and lumbar lordosis mismatch; TPA, T1 pelvic angle; T1SPI, T1 spinopelvic inclination; SVA, sagittal vertical axis.

*A statistically significant difference in parameters between postoperatively and at the final follow-up (*P *< 0.05).

**A statistically significant difference in parameters between preoperatively and postoperatively (*P *< 0.01).

### Relationship between postoperative immediate parameters and final follow-up parameters

**Figure 2:**
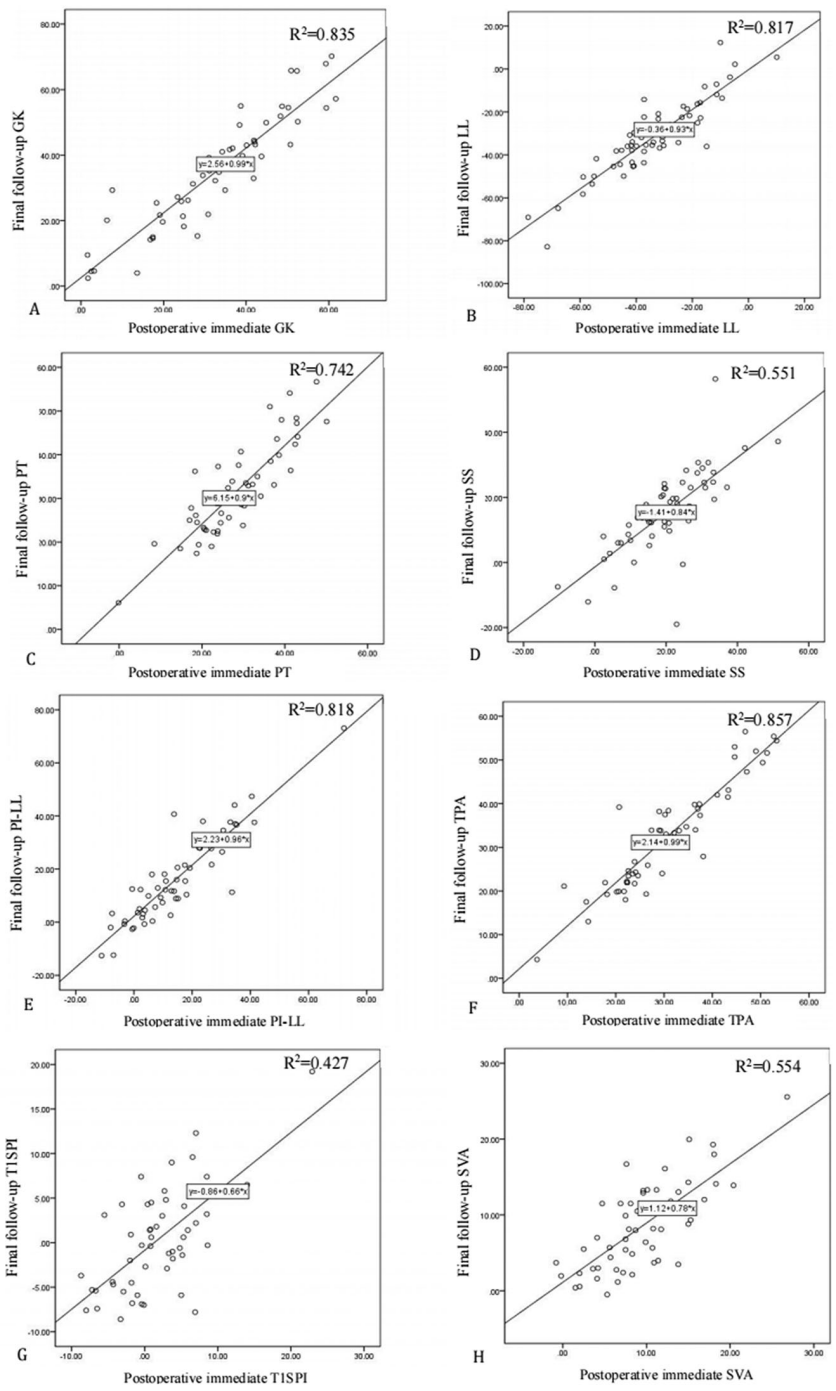
Linear regression analysis of postoperative immediate parameters and final follow-up parameters. (**A**) Final follow-up global kyphosis (GK) = 2.56 + 0.99 × postoperative immediate GK, *R*^2 ^= 0.835; (**B**) final follow-up lumbar lordosis (LL) = −0.36 + 0.93 × postoperative immediate LL, *R*^2 ^= 0.817; (**C**) final follow-up pelvic tilt (PT) = 6.15 + 0.9 × postoperative immediate PT, *R*^2 ^= 0.742; (**D**) final follow-up sacral slope (SS) = −1.41 + 0.84 × postoperative immediate SS, *R*^2 ^= 0.551; (**E**) final follow-up pelvic incidence and lumbar lordosis mismatch (PI–LL) = 2.23 + 0.96 × postoperative immediate PI–LL, *R*^2 ^= 0.818; (**F**) final follow-up T1 pelvic angle (TPA) = 2.14 + 0.99 × postoperative immediate TPA, *R*^2 ^= 0.857; (**G**) final follow-up T1 spinopelvic inclination (T1SPI) = −0.86 + 0.66 × postoperative immediate T1SPI, *R*^2 ^= 0.427; and (**H**) final follow-up sagittal vertical axis (SVA) = 1.12 + 0.78 × postoperative immediate SVA, *R*^2 ^= 0.554.

Linear regression analysis showed that the postoperative immediate GK, LL, PT, SS, PI–LL, TPA, T1SPI, and SVA were positively correlated with the corresponding parameters at the final follow-up (*R*^2 ^= 0.835, 0.817, 0.742, 0.551, 0.818, 0.857, 0.427, and 0.554, respectively, all *P *< 0.001, Figure 2). All these parameters at the final follow-up could be predicted with the immediate postoperative parameters through their linear regression equation.

### Correlation between postoperative immediate parameters and clinical outcome

At the final follow-up, 29 patients (37.7%) had ODI ≤ 20 and 32 patients (41.6%) had SRS-22 ≥ 4.0, which were considered to have achieved a good clinical outcome. All domains of the SRS-22 and ODI were significantly improved at the final follow-up (*P *< 0.05) (Table 2). The correlation between the immediate postoperative parameters and the final follow-up clinical outcomes (ODI and SRS-22) was examined. The results showed that the postoperative immediate PI, SS, T1SPI, and SVA values significantly correlated with the total ODI (all *P *< 0.05), while the postoperative immediate T1SPI, SVA, and TPA values significantly correlated with the total SRS-22 score (all *P* < 0.05) (Table 3). This means that the clinical outcomes at the final follow-up could be assessed and predicted with the immediate postoperative parameters.

**Table 2:** Clinical outcomes of Oswestry Disability Index and Scoliosis Research Society-22 score after surgery.

Items	Preoperative	Final follow-up	Improvement	*P-*value
ODI-walking	1.72 ± 1.21	0.66 ± 1.02	1.06 ± 1.46	<0.001*
ODI-sitting	1.40 ± 1.10	1.04 ± 0.76	0.36 ± 1.26	0.043*
ODI-standing	2.34 ± 1.27	0.94 ± 0.93	1.40 ± 1.35	<0.001*
Total ODI	40.02 ± 18.20	21.45 ± 11.85	18.57 ± 20.45	<0.001*
SRS-22-pain	3.28 ± 0.91	3.89 ± 0.72	0.61 ± 0.91	<0.001*
SRS-22-function	2.80 ± 0.85	3.41 ± 0.63	0.60 ± 0.79	<0.001*
SRS-22-appearance	2.00 ± 0.71	3.88 ± 0.64	1.87 ± 0.91	<0.001*
SRS-22-mental health	2.93 ± 0.87	3.98 ± 0.77	1.05 ± 0.91	<0.001*
SRS-22-satisfaction	2.61 ± 0.90	4.42 ± 0.59	1.81 ± 1.06	<0.001*
Total SRS-22	2.62 ± 0.60	3.98 ± 0.44	1.36 ± 0.66	<0.001*

ODI, Oswestry Disability Index; SRS-22, Scoliosis Research Society-22.

*A statistically significant difference between preoperatively and at the final follow-up (*P *< 0.05).

**Table 3:** Correlation between postoperative immediate parameters and final follow-up Oswestry Disability Index and Scoliosis Research Society-22 scores.

Items	GK	PT	PI	SS	LL	PI–LL	TPA	T1SPI	SVA
ODI-walking	0.184	0.073	0.466**	0.440**	−0.238	0.107	0.177	0.231	0.184
ODI-sitting	0.277*	0.016	0.304*	0.314*	−0.269*	−0.047	0.081	0.132	0.106
ODI-standing	0.139	0.158	0.232	0.110	0.038	0.211	0.206	0.136	0.130
Total ODI	0.208	0.085	0.364**	0.326**	−0.238	−0.029	0.240	0.340*	0.330*
SRS-22-pain	−0.223	−0.164	−0.116	0.021	−0.068	−0.155	−0.286*	−0.297*	−0.335*
SRS-22-function	−0.216	−0.197	−0.247	−0.091	0.068	−0.115	−0.373**	−0.414**	−0.424**
SRS-22-appearance	−0.046	0.014	−0.023	−0.036	0.001	−0.017	−0.123	−0.269	−0.249
SRS-22-mental health	−0.254	−0.119	−0.100	−0.002	0.088	0.016	−0.178	−0.150	−0.183
SRS-22-satifacton	0.044	−0.039	−0.215	−0.199	0.148	−0.010	−0.128	−0.191	−0.183
Total SRS-22	−0.204	−0.143	−0.189	−0.076	0.062	−0.078	−0.301*	−0.360**	−0.377**

GK, global kyphosis; PT, pelvic tilt; PI, pelvic incidence; SS, sacral slope; LL, lumbar lordosis; PI–LL, pelvic incidence and lumbar lordosis mismatch; TPA, T1 pelvic angle; T1SPI, T1 spinopelvic inclination; SVA, sagittal vertical axis; ODI, Oswestry Disability Index; SRS-22, Scoliosis Research Society-22.

*A statistically significant correlation (*P *< 0.05).

**A statistically significant correlation (*P *< 0.01).

### Optimal thresholds of the clinically relevant parameters

**Figure 3:**
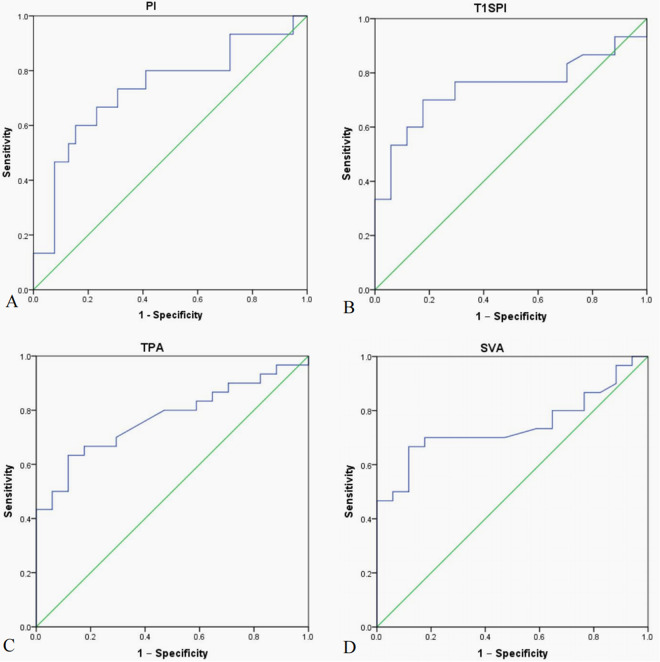
Receiver operating characteristic (ROC) curve analysis of clinically relevant parameters for the optimal threshold value. (**A**) The area under curve (AUC) for pelvic incidence (PI) was 0.733, the optimal threshold of PI was ≤49.2° for obtaining good clinical outcome with sensitivity of 73.3% and false-positive rate (1-Specificity) of 30.8%. (**B**) The AUC for T1 spinopelvic inclination (T1SPI) was 0.746, and the optimal threshold of T1SPI was ≤0.9° for obtaining a good clinical outcome with a sensitivity of 70.0% and a false-positive rate of 17.6%. (**C**) The AUC for T1 pelvic angle (TPA) was 0.772, and the optimal threshold of TPA was ≤31.5° for obtaining good clinical outcome with a sensitivity of 63.3% and a false-positive rate of 11.8%. (**D**) The AUC for sagittal vertical axis (SVA) was 0.741, and the optimal threshold of SVA was ≤9.3 cm for obtaining good clinical outcome with a sensitivity of 66.7% and a false-positive rate of 11.8%.

The clinically relevant parameters (PI, SS, T1SPI, SVA, and TPA) were subjected to ROC curve analysis to determine the optimal thresholds for obtaining good clinical outcomes. With total ODI as a state variable, only the PI showed a statistical significance (*P *< 0.05), while the SS, T1SPI, and SVA showed no significant difference (all *P *> 0.05). The optimal value of PI was ≤49.2° for obtaining good clinical outcome with a sensitivity of 99.8% and a false-positive rate of 36.7% (Figure 3A). With total SRS-22 as a state variable, all of the postoperative immediate T1SPI, TPA, and SVA were significantly different (all *P *< 0.05). The optimal value of T1SPI was ≤0.9° for obtaining good clinical outcome with a sensitivity of 70.0% and a false-positive rate of 17.6% (Figure 3B). The optimal value of TPA was ≤31.5° for obtaining good clinical outcome with a sensitivity of 63.3% and a false-positive rate of 11.8% (Figure 3C). The optimal value of SVA was ≤9.3 cm for obtaining good clinical outcome with a sensitivity of 63.3% and a false-positive rate of 11.8% (Figure 3D).

### Identifying the key clinically relevant parameters for total SRS-22

Multiple stepwise linear regression analysis was performed to determine the important clinically relevant parameters. With the total SRS-22 score as the dependent variable, the postoperative immediate T1SPI, TPA, and SVA were entered into the analysis. Finally, the SVA was the only parameter included in the regression model. The linear regression equation was total SRS-22 score = 4.247–0.033 × postoperative immediate SVA (adjusted *R*^2 ^= 0.125, *P *= 0.006), which indicates that the model explains 12.5% of the variability in the cohort (Table 4).

**Table 4:** Multiple stepwise linear regression analysis for the key clinically relevant parameter with total Scoliosis Research Society-22 as the dependent variable.

Variable	*B*	Standard error	Standardized beta coefficient	*t*	*P-*value
(Constant)	4.247	0.131		32.359	0.000
Postoperative immediate SVA	−0.033	0.012	−0.377	−2.878	0.006

With adjusted *R*^2 ^= 12.5%.

SVA, sagittal vertical axis.

### Comparing differences between groups with different pelvic incidences

**Figure 4:**
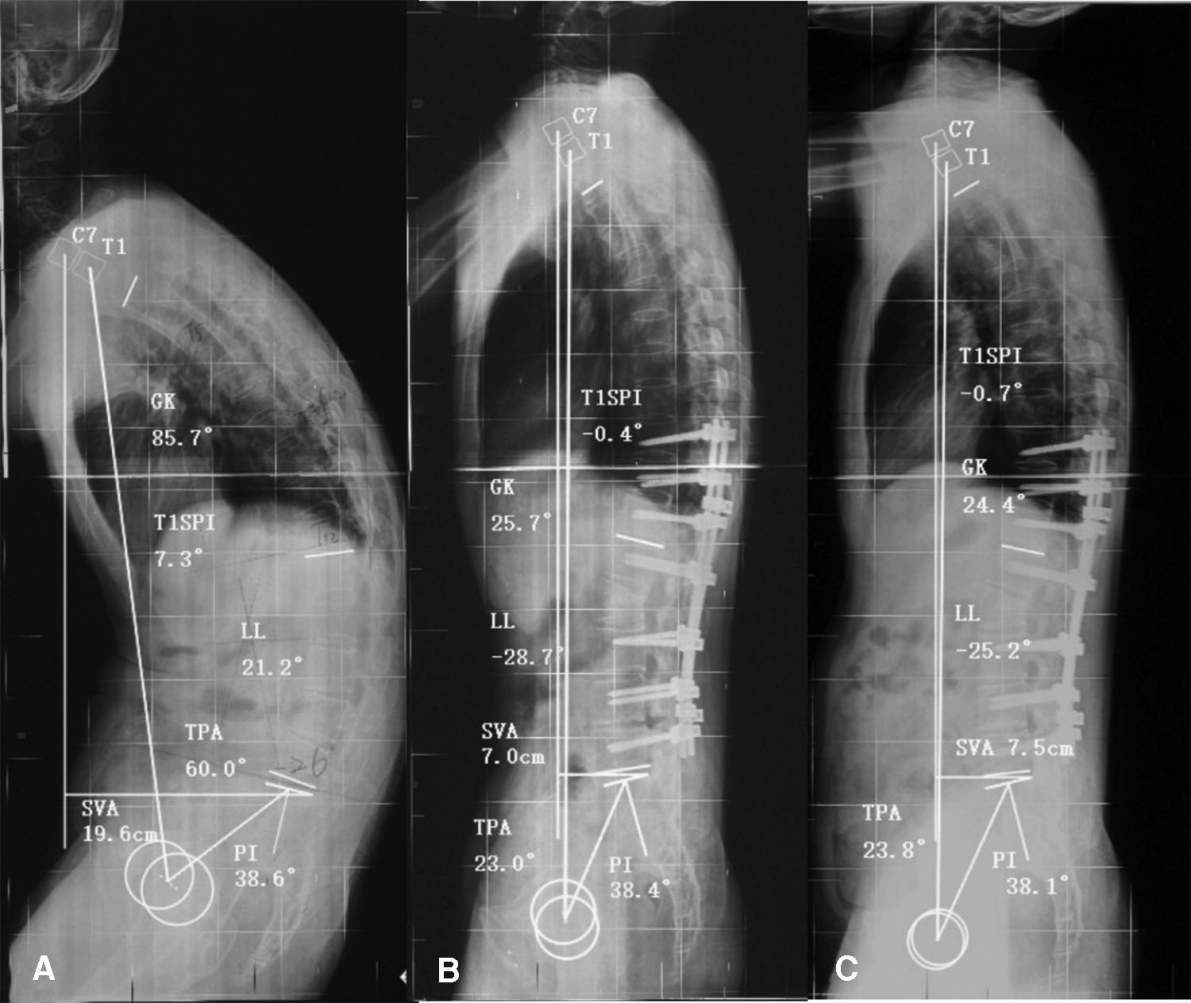
A 37-year-old man with ankylosing spondylitis kyphosis for 11 years. (**A**) The preoperative sagittal parameters were pelvic incidence (PI) = 38.6°, T1 spinopelvic inclination (T1SPI) = 7.3°, T1 pelvic angle (TPA) = 60.0°, and sagittal vertical axis (SVA) = 19.6 cm. (**B**) After L2 corrective osteotomy, the immediate postoperative sagittal parameters were PI = 38.4° (<49.2°), T1SPI = −0.4° (<0.9°), TPA = 23.0° (<31.5°), and SVA = 7.0 cm (<9.3 cm), all of which met the optimal parameter threshold values. (**C**) At the 25-month follow-up, the correction was well maintained, with PI = 38.1°, T1SPI = −0.7°, TPA = 23.8°, and SVA = 7.5 cm. The patient presented with a favorable health-related quality of life (total Oswestry Disability Index (ODI) = 0, total Scoliosis Research Society-22 (SRS-22) = 4.85).

The cohort was divided into two groups according to the PI threshold of 49.2°. Forty-four patients with PI ≤ 49.2° were in group A, and 33 patients with PI* *>* *49.2° were in group B. Preoperatively, group A had smaller PI, PT, SS, PI–LL, T1SPI, and TPA than group B (*P *< 0.05); there was no significant difference in all domains of ODI and SRS-22 (*P *> 0.05). Postoperatively, the PI, PT, SS, LL, PI–LL, TPA, and SVA were smaller in group A than in group B (*P *< 0.05). The average values of postoperative immediate T1SPI, TPA, and SVA in group A met the standard of optimal sagittal alignment (T1SPI* *≤* *0.85°, TPA* *≤* *31.5°, and SVA* *≤* *9.3 cm), while all of them were over the threshold values in group B. At the final follow-up, the total ODI was lower in group A than in group B (*P *< 0.05), while there was no significant difference in SRS-22 (*P *> 0.05). The incidence of sagittal imbalance at the final follow-up was also lower in group A than in group B (Table 5 and Figure 4).

**Table 5:** Differences in radiographic parameters and clinical outcomes with different pelvic incidences.

Variables	Group A (PI ≤ 49.2°, *n* = 44)	Group B (PI > 49.2°, *n* = 33)	*P-*value
Preoperative GK (°)	82.1 ± 19.6	88.6 ± 30.1	0.250
Preoperative PI (°)	40.5 ± 7.6	59.8 ± 11.3	<0.001*
Preoperative PT (°)	35.2 ± 9.8	43.2 ± 12.2	0.002*
Preoperative SS (°)	5.4 ± 10.3	16.8 ± 12.9	<0.001*
Preoperative LL (°)	9.0 ± 20.8	1.8 ± 22.8	0.158
Preoperative PI–LL (°)	49.5 ± 21.4	59.6 ± 21.2	0.044*
Preoperative T1SPI (°)	14.9 ± 14.5	23.1 ± 18.5	0.032*
Preoperative TPA (°)	51.4 ± 17.9	66.4 ± 17.7	<0.001*
Preoperative SVA (cm)	21.2 ± 8.5	25.2 ± 9.1	0.057
Preoperative ODI-walking	1.79 ± 1.10	1.64 ± 1.35	0.667
Preoperative ODI-sitting	1.29 ± 1.12	1.52 ± 1.08	0.443
Preoperative ODI-standing	2.36 ± 1.34	2.32 ± 1.22	0.917
Preoperative total ODI	37.65 ± 20.65	42.37 ± 19.81	0.400
Preoperative SRS-22-pain	3.39 ± 0.93	3.16 ± 0.89	0.373
Preoperative SRS-22-function	2.94 ± 0.97	2.66 ± 0.67	0.224
Preoperative SRS-22-appearance	1.99 ± 0.79	2.01 ± 0.61	0.910
Preoperative SRS-22-mental health	2.86 ± 1.07	3.00 ± 0.61	0.567
Preoperative SRS-22-satisfaction	2.82 ± 0.93	2.38 ± 0.82	0.073
Preoperative total SRS-22	2.80 ± 0.70	2.64 ± 0.50	0.356
Postoperative immediate GK (°)	34.2 ± 14.7	32.8 ± 15.5	0.700
Postoperative immediate PI (°)	40.6 ± 6.7	59.3 ± 8.8	<0.001*
Postoperative immediate PT (°)	25.3 ± 9.1	32.1 ± 9.2	0.002*
Postoperative immediate SS (°)	15.3 ± 9.6	26.8 ± 11.1	<0.001*
Postoperative immediate LL (°)	−29.5 ± 13.3	−40.6 ± 20.8	0.006*
Postoperative immediate PI–LL (°)	11.1 ± 12.5	18.3 ± 18.8	0.048*
Postoperative immediate T1SPI (°)	0.2 ± 5.1	2.6 ± 6.4	0.075
Postoperative immediate TPA (°)	25.8 ± 9.1	35.9 ± 12.3	<0.001*
Postoperative immediate SVA (cm)	7.9 ± 4.9	11.2 ± 5.9	0.011*
Final follow-up ODI-walking	0.30 ± 0.53	1.13 ± 1.26	0.006*
Final follow-up ODI-sitting	0.80 ± 0.55	1.29 ± 0.91	0.025*
Final follow-up ODI-standing	0.73 ± 0.69	1.21 ± 1.10	0.059
Final follow-up total ODI	19.29 ± 11.29	28.69 ± 15.10	0.012*
Final follow-up SRS-22-pain	4.03 ± 0.60	3.73 ± 0.83	0.143
Final follow-up SRS-22-function	3.52 ± 0.58	3.27 ± 0.66	0.139
Final follow-up SRS-22-appearance	3.92 ± 0.65	3.80 ± 0.64	0.490
Final follow-up SRS-22-mental health	4.03 ± 0.82	3.92 ± 0.71	0.607
Final follow-up SRS-22-satisfaction	4.53 ± 0.58	4.29 ± 0.59	0.138
Final follow-up total SRS-22	4.01 ± 0.46	3.80 ± 0.51	0.122
Incidence of sagittal imbalance at the final follow-up	56.8% (25/44)	81.8% (27/33)	0.020*

GK, global kyphosis; PI, pelvic incidence; PT, pelvic tilt; SS, sacral slope; LL, lumbar lordosis; PI–LL, pelvic incidence and lumbar lordosis mismatch; T1SPI, T1 spinopelvic inclination; TPA, T1 pelvic angle; SVA, sagittal vertical axis. ODI, Oswestry Disability Index; SRS-22, Scoliosis Research Society-22.

*A statistically significant difference between group A and group B (*P *< 0.05).

## Discussion

Sagittal realignment in AS patients after the corrective osteotomy is a primary determinant of clinical outcome measures and is a complex challenge for surgeons (4, 7, 8). Failure to achieve optimal immediate sagittal alignment might result in residual kyphosis, increasing the risk of sagittal imbalance, instrumental failure, and even reoperation (8). However, until now, few studies have explored the immediate postoperative sagittal alignment in AS patients with kyphosis after corrective osteotomy. The goals for AS kyphosis correction are still unclear, which seriously limits preoperative planning and impairs postoperative clinical outcomes.

In this study, correction loss occurred in GK, LL, PT, SS, TPA, and T1SPI at the final follow-up. However, all the final follow-up parameters linearly correlated with their immediate postoperative parameters. This meant that sagittal alignment at the mid- or long-term follow-up could be evaluated and predicted with some parameters immediately after surgery and made it possible to intervene to prevent severe correction loss early. Furthermore, the postoperative immediate parameters were also significantly correlated with the clinical outcome (ODI and SRS-22) at the final follow-up. Among them, the pelvic parameters (PI and SS) and sagittal global parameters (T1SPI, TPA, and SVA) were closely correlated with ODI and/or SRS-22 scores, which were also consistent with the findings reported by Schwab and Lafage (3, 9). The results also revealed that the reconstruction of sagittal realignment (T1SPI, TPA, and SVA) and unique parameters (PI) could be used to assess and predict the clinical outcome measures at the final follow-up. The ROC analysis indicated that when postoperative immediate T1SPI* *≤* *0.9°, TPA* *≤* *31.5°, SVA* *≤* *9.3 cm, and PI* *≤* *49.2°, the AS patients were more likely to obtain a good clinical outcome at the final follow-up.

The T1SPI reflects the position of T1 relative to the pelvis through the hips. This parameter might be more pragmatic and accurate than the SVA in noncalibrated radiographs. In this study, the optimal T1SPI for achieving a satisfactory clinical outcome was ≤0.9°, while the normative value was −1.4° ± 2.7° (10). This means that corrective osteotomy should be performed to keep AS patients with a relatively low T1SPI to achieve a good clinical outcome because a large T1SPI might displace the trunk anteriorly relative to the femoral heads, resulting in malpositioning of the trunk in terms of its gravity and causing pain and disability (9). However, few studies have focused on the influence of T1SPIs on AS patients until now.

The goal for postoperative SVA varies among previous studies. Kim et al. (11) reported that the maintenance of SVA < 8 cm was important for ultimate sagittal reconstruction in fixed sagittal imbalance. Van Royen et al. (12) reported that the postoperative SVA ideally ranged from 5 cm to 10 cm, while Schwab et al. (13) reported that a postoperative SVA of more than 10 cm could be considered to indicate failed realignment. In this study, the optimal SVA was ≤9.3 cm for a good clinical outcome. In patients with ankylosed cervical vertebrae, sagittal alignment cannot be corrected perfectly to be within a normal range because a chin-brow vertical angle in the range of 10°–20° needs to be ensured and horizontal vision needs to be maintained for patients postoperatively (14). Meanwhile, although there may be some residual deformity in these patients, it might not affect their ability to perform basic daily tasks, and the corrections are maintained well over the follow-up period. Therefore, it might not be necessary to correct the SVA to a normal value with excessive expanding operations, placing these patients at an increased risk for various surgical complications. Thus, an immediate postoperative SVA of 9.3 cm or less might be sufficient for the correction of severe kyphosis in AS.

Moreover, the postoperative SVA was found to be a key clinically relevant parameter by multiple linear regression analysis, which is in agreement with the findings of previous studies (9, 13). A predictive model of the postoperative SRS-22 score was calculated, i.e., total SRS-22 score = 4.247–0.033 × postoperative immediate SVA. This equation allows the follow-up total SRS-22 score to be predicted in AS patients, whereas only ODI predictive methods have been reported in previous studies (4, 7, 13).

Unlike the SVA, the TPA is an angle reflecting global sagittal alignment, which does not vary on the basis of pelvic retroversion or patient standing posture (15, 16). To date, the optimal TPA for AS patients after the corrective osteotomy is still unclear. Protopsaltis et al. (15) found that TPA > 20° might result in severe disability (ODI > 40) for ASD, and they recommended TPA < 14° as a postoperative target (ODI < 20). However, Banno et al. (16) investigated 656 elderly healthy volunteers and determined that a TPA of 26° was the threshold value for an ODI of 40, while a TPA of 20° was the threshold value for an ODI of 20. Their study also noted that the optimal TPA varies widely by race, sex, and disease (16). Recently, Huang et al. (4) reported TPA < 22° as a goal in AS patients to achieve a satisfactory clinical outcome (ODI < 20) following osteotomy. According to our data, the optimal TPA was <31.5°, which was calculated on the basis of a total SRS-22 score ≥4.0. Several reasons could explain the difference between this optimal TPA and that reported by Huang et al. (4). First, the patients in this study had more severe deformities than those in the study by Huang and were thus hard to correct perfectly but still satisfied with the improvement in quality of life. Second, the optimal threshold value for the TPA in this study was calculated by ROC curve analysis based on a total SRS-22 score ≥4.0, while Huang used linear regression equations to calculate the TPA based on ODI <20.

PI is a constant morphological parameter that is unique regardless of the rotation of the pelvis (7, 8, 17). Our data indicated that PI plays a key role in sagittal alignment reconstruction and significantly affects clinical outcome measures. Although PI showed no change after surgery, it was highly correlated with the recovery of clinically relevant parameters (1, 8, 17). For patients with a large PI value, it is difficult to achieve spinal and pelvic balance, leading to a negative clinical outcome and potentially even large correction loss at the final follow-up (7, 18, 19). Qian et al. (8) reported that patients with PI ≤ 50° were more likely to achieve spinopelvic matching and decrease the chance of sagittal imbalance on follow-up. Similar to the study by Qian et al. (8), this study validated the conclusion that AS patients with PI ≤ 49.2° achieved optimal immediate postoperative sagittal alignment and obtained better clinical outcomes and a lower incidence of sagittal imbalance than those with PI* *>* *49.2° at the final follow-up. These results further confirmed that PI is a critical parameter for sagittal realignment that affects global balance achievement and clinical outcome restoration in AS patients after osteotomy. Therefore, the surgeon should pay more attention to PI in reconstructing sagittal alignment to achieve optimal sagittal alignment and should choose appropriate osteotomy techniques and the number of osteotomy segments to obtain enough correction for correction (3, 19).

Of the four key radiographic parameters determined in this study, the T1SPI, TPA, and SVA represent spinal alignment and can be corrected directly with surgical treatment; although PI, as a pelvic parameter, cannot be changed by surgery, it can influence the spine and pelvis harmony, affecting the maintenance of spinopelvic balance. Therefore, the spinal and pelvic parameters should be taken into consideration for a good clinical outcome while reconstructing an optimal sagittal alignment.

### Limitations

First, this was a retrospective study in which a limited number of patients were enrolled. Second, the influence of one-level and two-level osteotomy on the postoperative sagittal alignment was not compared separately. Third, radiographic parameter evaluation is only one aspect in evaluating the clinical outcome in AS patients. The surgery itself might influence the clinical outcome, and other potential factors, such as age, sex, and comorbidities, might also affect the clinical outcome reported by patients. Finally, although most of the AS patients who underwent osteotomy were in an inflammatory static state, AS did affect the quality of life on follow-up. In the future, a prospective study with a larger sample size is required to further confirm the conclusions.

## Conclusion

The results of this study demonstrated that the immediate postoperative parameters could be used to evaluate and predict the final follow-up parameters and clinical outcome in AS patients. In particular, the PI and postoperative immediate T1SPI, TPA, and SVA significantly correlated with the clinical outcome measures. The optimal postoperative immediate sagittal alignment was T1SPI* *≤* *0.9°, TPA* *≤* *31.5°, and SVA* *≤* *9.3 cm, providing a reference for kyphosis correction and a means for clinical outcome evaluation. Patients with lower PI values (≤49.2°) are more likely to achieve better sagittal alignment and clinical outcomes after corrective osteotomy.

## Data Availability

The raw data supporting the conclusions of this article will be made available by the authors, without undue reservation.
